# An Ensemble Learning Aided Computer Vision Method with Advanced Color Enhancement for Corroded Bolt Detection in Tunnels

**DOI:** 10.3390/s22249715

**Published:** 2022-12-11

**Authors:** Lei Tan, Tao Tang, Dajun Yuan

**Affiliations:** 1 State Key Laboratory of Rail Traffic Control and Safety, Beijing Jiaotong University, Beijing 100044, China; 2 Beijing Municipal Engineering Research Institute, Beijing 100037, China; 3 School of Electronic and Information Engineering, Beijing Jiaotong University, Beijing 100044, China; 4 School of Civil Engineering, Beijing Jiaotong University, Beijing 100044, China

**Keywords:** corroded bolt detection, computer vision, color enhancement, ensemble learning

## Abstract

Bolts, as the basic units of tunnel linings, are crucial to safe tunnel service. Caused by the moist and complex environment in the tunnel, corrosion becomes a significant defect of bolts. Computer vision technology is adopted because manual patrol inspection is inefficient and often misses the corroded bolts. However, most current studies are conducted in a laboratory with good lighting conditions, while their effects in actual practice have yet to be considered, and the accuracy also needs to be improved. In this paper, we put forward an Ensemble Learning approach combining our Improved MultiScale Retinex with Color Restoration (IMSRCR) and You Only Look Once (YOLO) based on truly acquired tunnel image data to detect corroded bolts in the lining. The IMSRCR sharpens and strengthens the features of the lining pictures, weakening the bad effect of a dim environment compared with the existing MSRCR. Furthermore, we combine models with different parameters that show different performance using the ensemble learning method, greatly improving the accuracy. Sufficient comparisons and ablation experiments based on a dataset collected from the tunnel in service are conducted to prove the superiority of our proposed algorithm.

## 1. Introduction

Railway transportation has become the main mode of land transport with its remarkable carrying capacity and fast speed [1,2]. As an important branch, subway systems have developed rapidly in recent years [3], becoming the preferred traveling way for city dwellers. The lining, which is fixed and arranged by bolts, supports the tunnel structure and guarantees the operation of metros. However, the bolts are exposed to the open air, usually influenced by moisture and air pollutants, and the steel material thus tends to become corroded [4,5,6]. When it comes to maintenance and repair, human-based visual inspection still dominates the tunnel industry, which is also limited by training level. Patrol inspectors have to check all bolts during non-running times such as night and early morning. However, commonly, an inspection team composed of 10 to 15 trained maintainers could check two to three kilometers during a maintenance period of about three hours, which is costly and inefficient. Besides, quite a few bolts are misdiagnosed as normal or corroded due to the poor light in tunnels and fatigue caused by night work. Hence, some researchers have tried many approaches to design an automatic, high-accuracy, and fast detection speed method for practical engineering projects.

Computer Vision (CV), which overcomes the limitations of visual inspection by trained human resources and the ability to detect structural damage in images remotely [7,8], has become a prioritized technique for corroded bolt detection. However, the traditional CV algorithms require the manual design of filter modules, which has poor robustness and low accuracy. Deep learning-based CV bolt corrosion detection becomes available for engineering as deep learning develops [9,10,11]. For instance, Cha et al. [12] developed an autonomous structural visual inspection method via Region-based Convolutional Neural Networks (RCNNs) for real-time damage detection covering concrete cracks, steel and bolt corrosion, and steel delamination. Ta et al. [13] monitored and identified the corrosion levels of corroded bolts in a lab-scale steel structure with good illumination using a Mask-RCNN. Suh et al. [14] adopted a Faster RCNN-based model to detect and locate damage types, including bolt corrosion. These RCNNs search the target area with selective search and generate nearly 2000 eigenvectors for each figure. They are mostly applied in the precise pixel-level detection task. However, it is not easy to deploy RCNN models in practical applications compared to end-to-end models. Plus, it is not necessary to precisely distinguish the target pixels on the corrosion bolt in practice at the expense of speed and cost. Another branch of deep learning target detection algorithms, You Only Look Once (YOLO), reinterprets the principle of object detection tasks from classifications to regressions, speeding up the training and detecting processes [15,16,17]. We select YOLOv5 nano (YOLOv5n) as the basis of our proposed model caused of its speed, end-to-end characteristics, and high precision compared with the two-stage detectors.

Although the YOLOv5n shows its superior performance in computing speed and resource consumption, the complex corrosion targets still require improvements in accuracy. Using multiple models with different preferences, ensemble learning makes a better and more comprehensive decision to avoid the wrong prediction created by weak classifiers. For example, Xu et al. [18] applied ensemble deep learning technology to learn and extract features of forest fires. Mohammad et al. [19] presented an ensemble deep-learning approach to recognize structural corrosion in drone images. Seijo-Pardo et al. [20] concluded ensemble learning of homogeneous and heterogeneous approaches, showing the availability of integrating models with different parameters. Inspired by these works, we put forward ensemble learning with YOLOv5n (YOLOv5n-EL) to raise accuracy without slowing down the computing speed too much.

In addition to the corroded bolt detector, tunnels are usually damp and dim, weakening the tunnel scan image to low definition, poor contrast, and color distortion. These problems bring big troubles to the task of corroded bolt detection in such tunnels, which require figures to be pre-processed to make the features of the image more apparent for better corroded bolt detection. It has been proved that the Retinex theory (a color-invariance-based principle) is effective for low-light image enhancement like night and underwater [21,22,23]. Retinex mainly consists of three basic algorithms—Single Scale Retinex (SSR), MultiScale Retinex (MSR), and MultiScale Retinex with Color Restoration (MSRCR). Compared with SSR and MSR, MSRCR shows better image quality improvement and the ability to avoid the color distortion caused by the imbalance of each color channel proportion after convolution computation. However, the performance still degrades in the dim tunnel environment caused by its Gaussian Blur, which reduces the sharpness of edges while brightening the dark areas. Thus, we proposed the Improved-MSRCR (IMSRCR) algorithm to solve the problem of fuzzy bolt edges in low-illumination tunnel images using auto-matched dynamic filters and $L_{0}$ regularization. Through a combination scheme of the IMSRCR and the YOLOv5n-EL, our model appears to have excellent performance at bolt corrosion detection. Our main contributions can be summarized as follows.

1. We optimized the MSRCR color enhancement algorithm based on auto-matched dynamic filters and $L_{0}$ regularization to avoid blurring the image when brightening the dark areas.
2. We put forward ensemble learning with its fusion strategy combining models with different parameters to improve precision accuracy.
3. The experiments are conducted on actual data collected from a practical railway tunnel. We disclosed our labeled dataset, the first public corroded lining bolt dataset using a professional tunnel scanner.

The rest of this paper is organized as follows. Section 2 exhaustively describes the proposed approach covering the improved color-enhanced module and ensemble learning algorithm for bolt corrosion detection. Section 3 thoroughly exhibits the details of the experiments, including the dataset, experiment settings, comparison schemes, performance evaluations, and the analysis of the results. Section 4 gives a discussion about the method. Section 5 outlines our main results.

## 2. Methodology

**Figure 1:**
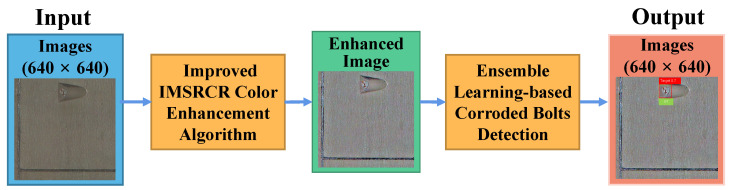
Flow chart of corroded bolt detection scheme in a dim tunnel.

Figure 1 depicts the flow chart of the corroded bolt detection scheme in a dim tunnel, including two main modules, i.e., the image color enhancement algorithm and the object detection module. Considering the difficulty of distinguishing corroded and normal bolts in a dim environment, an improved MSRCR (IMSRCR) is proposed to sharpen the contrast between the rust-infected area and the background, enhancing the appearance of image features. Then, for essential prediction speed and training efficiency in the object detection module, YOLOv5n is introduced to finish the object detection and location of corroded bolts on the color enhancement image, which is an end-to-end train and predict structure. For a further step up in accuracy, we propose YOLOv5n-EL based on YOLOv5n. Specifically, we train a series of models with different parameters and adopt ensemble learning to integrate all model outputs.

### 2.1. The Improved IMSRCR Color Enhancement Algorithm

As is well-known, the illumination is poor, so the tunnel images gathered are dim and unclear. Thus, we need to enhance the contrast between the bolts and the background. MSRCR is developed on MSR and SSR based on Retinex theory, which has been approved as an effective color enhancement method. However, MSRCR has a limited effect in dark areas and the edges of the dark areas. In our work, we propose IMSRCR to enhance the bolts features in dark areas. According to Retinex, the observed image I(x,y) can be divided into the reflection component R(x,y) carrying target information and the irradiation component L(x,y) of ambient light is

(1) I(x,y)=L(x,y)×R(x,y).

Therefore, image enhancement aims to get rid of the irradiated component and extract a reflective part that carries information about the object. By simple mathematical transformation, we can get the expression of R(x,y) with

(2) logR(x,y)=logI(x,y)−logL(x,y).

L(x,y) can be estimated through low-pass Gaussian center function F(x,y) and the observed image I(x,y) as

(3) logL(x,y)=log[F(x,y)⊗I(x,y)],

where F(x,y) is defined by

(4) $$F\left(x,y\right)=\lambda e^{-\frac{x^{2}+y^{2}}{2c^{2}}}.$$

Meanwhile, F(x,y) should satisfies

(5) ∫∫F(x,y)dxdy=1.

As a result, the expression of SSR can be obtained from (2)–(4) to

(6) $$r_{ssr}\left(x,y\right)=\log R\left(x,y\right)=\log I\left(x,y\right)-\log\left[F\left(x,y\right)⊗I\left(x,y\right)\right].$$

The parameter *c* in (4) is strongly related to the scale of image enhancement. However, the enhancements of SSR are not always satisfactory because the parameter *c* is not suitable for all kinds of images. In response to the above question, MSR imports Gaussian center function at different scales as

(7) $$r_{msr}\left(x,y\right)=\sum_{k}^{K}\omega_{k}\log I\left(x,y\right)-\log[F_{k}\left(x,y\right)⊗I\left(x,y\right)],$$

where $\omega_{k}$ and $F_{k}\left(x,y\right)$ meets the Equations (8)–(10).

(8) $$\sum_{k}^{K}\omega_{k}=1,$$

(9) $$F_{k}\left(x,y\right)=\lambda_{k}e^{-\frac{x^{2}+y^{2}}{2{c_{k}}^{2}}},$$

(10) $$\;\int \int \;F_{k}\left(x,y\right)dxdy=1.$$

Although MSR enhances image features at both low and high scales, color distortion will occur as the parameters are different for each color channel. Thus, the color recover factor *C* is added in MSRCR to keep the appearance true through

(11) $$r_{msrcr}\left(x,y\right)=C_{i}\sum_{k}^{K}\omega_{k}\log I_{i}\left(x,y\right)-\log\left[F_{k}\left(x,y\right)⊗I_{i}\left(x,y\right)\right],$$

where *i* represents the $i_{\mathrm{th}}$ color channel and $C_{i}$ can be expressed by

(12) $$\begin{array}{cc} C_{i} & =f[I_{i}^{\prime }\left(x,y\right)]\\ \; & =\beta \log[\alpha I_{i}^{\prime }\left(x,y\right)]\\ \; & =\beta \log[\alpha \frac{I_{i}\left(x,y\right)}{\sum_{j=1}^{N}I_{J}\left(x,y\right)}]\\ \; & =\beta \log[\alpha I_{i}\left(x,y)\right]-\beta \log\left[\sum_{j=1}^{N}I_{J}\left(x,y\right)\right], \end{array}$$

in which α denotes controlled nonlinear treatment strength and β is the gain constant.

**Figure 2:**
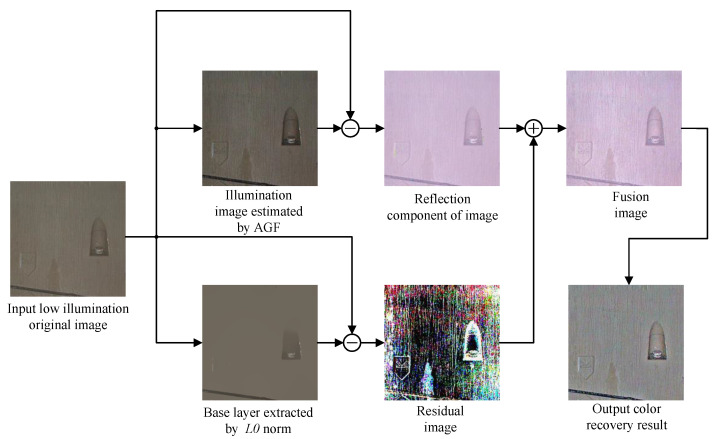
Flow path of IMSRCR.

Although MSRCR performs better in image enhancement comparing MSR and SSR, the edge of the enhanced image is still inconspicuous, which makes the performance of MSRCR degrade in a dim environment. Accordingly, we propose an IMSRCR algorithm to solve the problem of fuzzy bolt edges in low-illumination tunnel images. Our algorithm uses Automatic Guide Filtering (AGF) to estimate the illumination image first and then calculate the reflected image according to the Retinex theory mentioned above. Residual image is extracted by the norm. Finally, the color restoration is carried out on the fused image. The flow path of our algorithm is shown in Figure 2.

#### 2.1.1. Illumination Estimation

**Figure 3:**
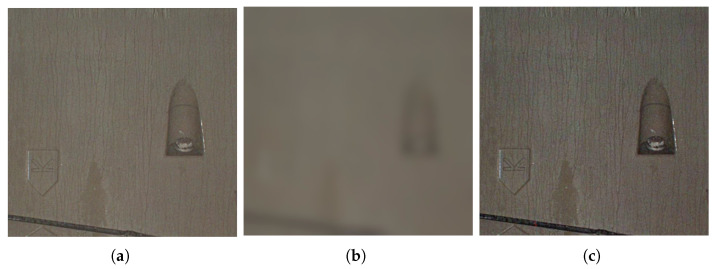
Original and illumination images. (**a**) Original image; (**b**) Illumination image estimated by Gaussian filter; (**c**) Illumination image estimated by AGF.

In order to reduce the edge blur problem of the Gaussian filter, the Illumination Estimation is powered by AGF, which is different from traditional MSRCR using a Gaussian filter. The illuminance images estimated by AGF and Gaussian filter are shown in Figure 3.

Guided filter is a local linear model with smooth edge preserving characteristics [24,25] which is defined as

(13) $$g_{t}=a_{k}\;G_{t}+b_{k}\;\;,\;\forall t\epsilon \Omega_{k},$$

where *g* is the output image after guided filtering and *G* is the guided image, $a_{k}$ and $b_{k}$ are the linear coefficients at the sub-windows $\Omega_{k}$, $\Omega_{k}$ represents the sub-window with scale *r*, and *t* is the index of pixels in $\Omega_{k}$. We specify to input image *I* as the guided image *Q*. $a_{k}$ and $b_{k}$ could be defined according to Guiding filtering-related theory as

(14) $$\begin{array}{cc} a_{k} & =\frac{\sigma_{k}^{2}}{\sigma_{k}^{2}+\varepsilon^{\prime }}\\ b_{k} & =\mu_{k}\left(1-a_{k}\right). \end{array}$$

The scale *r* of the guided filter is set to three values referring to the process of the MSRCR algorithm. The range of three values of scale *r* is $\left[1,r_{min}\right]$, $\left[r_{min},r_{mid}\right]$ and $\left[r_{mid},r_{max}\right]$ respectively [26]. $r_{min}$, $r_{mid}$ and $r_{max}$ could be determined as

(15) $$\begin{array}{cc} r_{min} & =\left\lfloor \frac{\min(m,\;n)}{2^{N}}\right\rfloor ,\\ r_{max} & =\left\lfloor \frac{\min(m,\;n)}{2}-1\right\rfloor ,\\ r_{mid} & =\left\lfloor \frac{r_{min}+r_{max}}{2}\right\rfloor , \end{array}$$

where *m* and *n* are the width and height of the image, and *N* is the number of selected scales. To balance the smoothing and edge-preserving effects of guided filtering, an Auto multi-scale selection algorithm is expressed by

(16) $$\begin{array}{cc} r_{1} & =\left\lfloor \frac{1+r_{min}}{2^{N}}\right\rfloor ,\\ r_{2} & =\left\lfloor \frac{r_{min}+r_{mid}}{2}\right\rfloor ,\\ r_{3} & =\left\lfloor \frac{r_{mid}+r_{max}}{2}\right\rfloor . \end{array}$$

The illumination estimation result applies AGF to each channel of the input image. The reflection component in the logarithmic domain could be defined according to the Retinex theory

(17) $$F_{AGF}=\sum_{j=1}^{3}\omega_{j}\left[logI_{i}\left(x,y\right)-logg_{i}\left(x,y\right)\right],$$

where $F_{AGF}$ is the reflected image channel corresponding to the AGF.

#### 2.1.2. Residual Fusion

**Figure 4:**
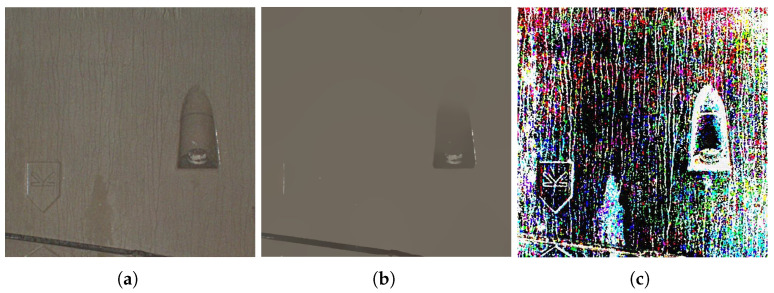
Original and residual images. (**a**) Original image; (**b**) Base layer extracted by $L_{0}$ norm; (**c**) Residual image.

In order to overcome the problem of $F_{AGF}$ detail loss, we used $L_{0}$ norm in IMSRCR [27]. Residual results extracted by $L_{0}$ norm is shown in Figure 4.

$L_{0}$ norm can be expressed as the number of non-zero elements in a vector. The $L_{0}$ norm of image gradient can be expressed as

(18) $$C\left(f\right):=\#\left\{p||f_{p}-f_{p+1}|\neq 0\right\},$$

where *p* and p+1 are adjacent elements in the image. $\left|f_{p}-f_{p+1}\right|$ is the image gradient which is the forward difference of the image. # represents the number of pixels in the image that satisfied $\left|f_{p}-f_{p+1}\right|\neq 0$. $C\left(f\right)$ is the $L_{0}$ norm of the image gradient.

Taking one-dimensional signal as an example, the objective function can be defined as

(19) $$\min_{f}\sum_{p}{\left(f_{p}-g_{p}\right)}^{2}\;\;s.t.\;\;C\left(f\right)=k.$$

It must be converted into unconstrained problems for two-dimensional images. We set smoothing parameter λ to 0.01 in combination with our use scene

(20) $$\min_{f}\sum_{p}{\left(f_{p}-g_{p}\right)}^{2}+\lambda \cdot C\left(f\right).$$

The number of gradients in the horizontal and vertical directions of the image needs to be constrained in the two-dimensional images. The objective function and its constraints are expressed as

(21) $$\begin{array}{c} \min_{f}\sum {\left(f_{p}-g_{p}\right)}^{2}+\lambda \cdot C\left(\partial_{x}f,\partial_{y}f\right),\\ C\left(\partial_{x}f,\partial_{y}f\right)=\#\left\{p∥\partial_{x}f_{p}\left|+\right|\partial_{y}f_{p}|\neq 0\right\}. \end{array}$$

Since the $L_{0}$ norm is non-differentiable, the variable splitting method is used here to relax it into two quadratic programming problems. Finally, the iterative method is used to find the global optimum. We rewrite the objective function as

(22) $$\begin{array}{c} \min_{f}\sum_{p}{\left(f_{p}-g_{p}\right)}^{2}+\lambda \cdot C\left(\partial_{x}f,\partial_{y}f\right)+\beta \cdot \sum^{p}\left({\left(\partial_{x}f_{p}-h_{p}\right)}^{2}+{\left(\partial_{y}f_{p}-v_{p}\right)}^{2}\right). \end{array}$$

The iterative solution result of the objective function is expressed as

(23) $$h_{p},v_{p}=\left\{\begin{array}{cc} \left(0,0\right) & {\left(\partial_{x}f_{p}\right)}^{2}+{\left(\partial_{y}f_{p}\right)}^{2}\leq \frac{\lambda }{\beta }\\ \left.\left(\partial_{x}f_{p},\partial_{y}f_{p}\right)\right. & \;\mathrm{otherwise}\; \end{array}\right.$$

**Figure 5:**
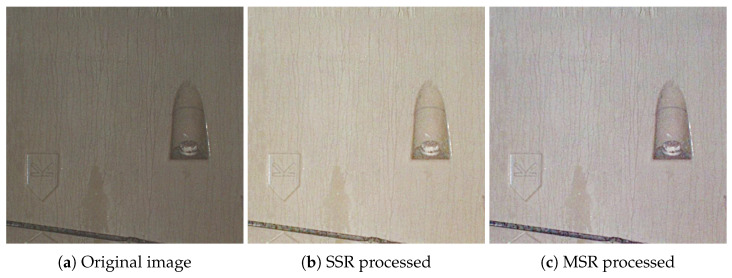

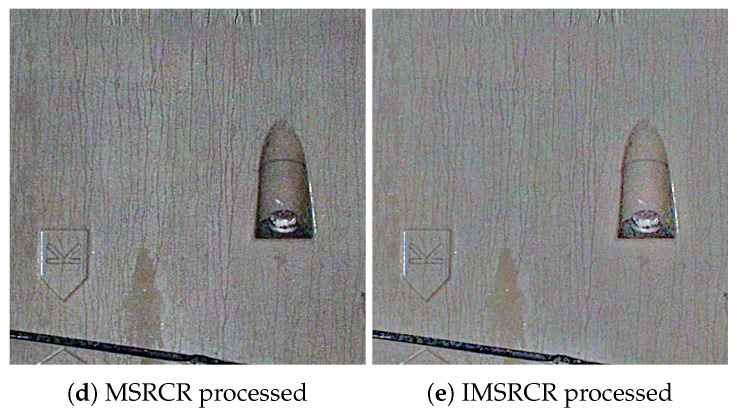
Effect comparison of different image enhancement algorithm.

As presented in Figure 5, the image processed by IMSRCR is more apparent and has higher color contrast based on subjective visual judgment. And the edge of the bolts is more clear compared with the enhanced image processed by SSR, MSR, and MSRCR. Hence, IMSRCR is developed for the detection module to ensure that the pictures inputted to YOLOv5n have distinct visual features.

### 2.2. Ensemble Learning-Based Corroded Bolts Detection

CV modules with different stages, mainly one and two, are used for object detection tasks. One-stage end-to-end algorithms give the prediction results (type and location) directly through the backbone, while two-stage methods form a series of sample boxes first, then classify and locate the object inside the boxes. So the non-end-to-end structure requires much more time than the one-stage method to train and detect separately, slowing the speed in real corroded bolt detection work. YOLOv5n, a fast and accurate one-stage CV model, is chosen as the baseline of our ensemble learning.

#### 2.2.1. Ensemble Learning Method

Usually, a target detection task is based on one given model to train and learn for a good performance in detection results. As far as we know, there are some excellent models to resolve the detection task, such as YOLO and FCNN. However, the performance of the models mentioned above can still be improved. Adjusting HyperParameters of training is a common technique to improve the model performance. However, it has a limited effect as the structure of the model restricts a better performance. Ensemble learning is a machine learning method that integrates the prediction of multiple deep learning models to improve robustness and detection performance. It processes the multiple model outputs as a decision question. If a mistake occurs on one of the multiple models and the others are right, the final output of ensemble learning will correct the error considering the whole model’s outputs. Compared with the single model, ensemble learning combining multiple models will improve the accuracy heavily.

**Figure 6:**
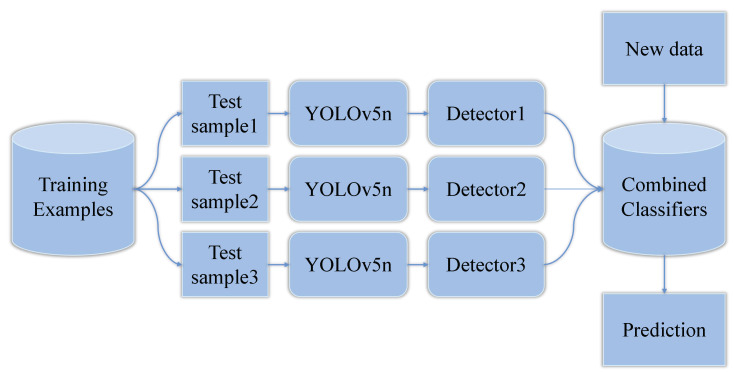
Structure of Ensemble Learning.

Ensemble learning can be divided into two categories according to training methods: Boosting and Bagging. Boosting constructs a series of object detectors through serial learning, which means the new detector is improved based on the adjustment to the mistake detection data weight in the last detector. In contrast, Bagging is a parallel learning method that utilizes the independence of different detectors to improve performance, while a single detector cannot extract whole features. In our work, Bagging is adopted as the ensemble learning method while we integrate different kinds of models which are independent of each other. The structure of ensemble learning is shown in Figure 6. It is worth noticing that our proposed integrated learning model is a parallel structure, corresponding to the use of multi-threaded parallel learning operations to avoid bringing excessive consumption of model training and inference time.

Bagging draws training data from the whole dataset at random and the drawn training data will be put back before the next round of extraction. This process will be continued for *k* rounds, so we can get *k* independent sub-datasets. Every sub-dataset is adopted to train a basic model. As a result, we can get *k* independent basic models.

#### 2.2.2. Fusion Strategy in Ensemble Learning

Fusion strategy is fundamental in ensemble learning. With an excellent fusion strategy, ensemble learning can combine the strengths of each model and get a better result comparing any single model without ensemble learning. We adopt a probabilistic ensemble method to combine the independent basic models in our work. Assume that we have an object with a label *y* and two outputs of the basic models $x_{1}$ and $x_{2}$ ( it can easily be expanded to more outputs ). As Bagging mentioned above, the basic models are independent, so the measurements are also conditionally independent, which can be formulated as

(24) $$p\left(x_{1},x_{2}|y\right)=p\left(x_{1}|y\right)p\left(x_{2}|y\right).$$

This is also can be expressed as $p\left(x_{1}|y\right)=p\left(x_{1}|x_{2},y\right)$ as the independence between $x_{1}$ and $x_{2}$ exists, which means that the $x_{2}$ will not be changed if we give the value of *y*. Our purpose is to get the value of *y*, which can be expressed as

(25) $$p\left(x_{1},x_{2}|y\right)=\frac{p\left(x_{1}|x_{2},y\right)p\left(y\right)}{p(x_{1},x_{2})}\propto p\left(x_{1}|x_{2},y\right)p\left(y\right).$$

As the independence mentioned above, the probabilistic relation can be written as

(26) $$p\left(y|x_{1},x_{2}\right)\propto p\left(x_{1}|y\right)p\left(x_{2}|y\right)p\left(y\right)\propto \frac{p\left(x_{1}|y\right)p\left(y\right)p\left(x_{2}|y\right)p\left(y\right)}{p(y)}\propto \frac{p\left(y|x_{1}\right)p\left(y|x_{2}\right)}{p(y)}.$$

Utilizing the probabilistic relation, we can calculate the score of *y*. Given the existence of conditional independence, it can be considered the optimal fusion scheme. The calculation can be formulated as

(27) $$p\left(y|{\left\{x_{i}\right\}}_{i=1}^{M}\right)\propto \frac{\prod_{i=1}^{M}p\left(y|x_{i}\right)}{p{\left(y\right)}^{M-1}}.$$

The class prior p(y) can be easily obtained by taking the statistics for *y* from the dataset. Then, according to (27), the results of all basic models can be fused.

## 3. Experiment

### 3.1. Data Acquisition System and Dataset

**Figure 7:**
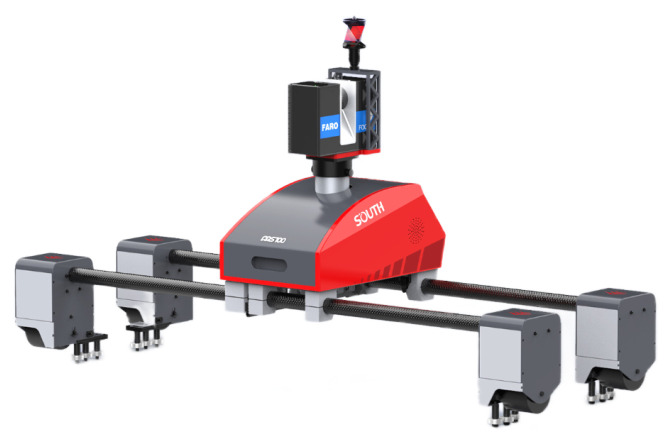
MS100 3D scanner.

Figure 7 shows the data acquisition system named MS100 produced by South Surveying & Mapping Technology Co., Ltd. (Guangzhou, China). It can automatically move and scan with a motor at a speed of 1 km/h in disease-scanning mode. The experiments are performed on the corroded bolt dataset collected from a certain Beijing metro tunnel in service. The dataset consists of 1441 pictures in the size 640×640. All the targets are labeled with a 100×100 ground truth box. We split the dataset into the training set and the test set in a ratio of 8:2, with the test set also serving as the validation set.

### 3.2. Experiment Settings

Experiments in the study have been implemented on an Intel${}^{®}$ Core${}^{\mathrm{TM}}$ i7-11700K CPU (3.6 GHz, 32 GB RAM) and an NVIDIA GeForce RTX 3060 (CUDA version 11.6) with Python 3.9.12 (PyTorch 1.11.0) in 64 Bit Ubuntu 18.04.1 Long Term Support operating system.

To train the module properly, we set the input resolution to 640×640 and use Stochastic Gradient Descent (SGD) with 0.9 momenta as the optimizer. The learning rate is initialized to 0.001 and the cosine decay with warm-up is selected as the learning rate schedule. All models have been trained completely in the experiments.

As for data augmentation, we set the image rotation rate to 0.5 and the image translation rate to 0.1. Both the image scale rate and image shear rate are set to 0.5. We mainly used Mosaic to further enhance the performance of the detector, and the Mosaic rate is set to 1.0.

### 3.3. Evaluation Metrics

Taking the popular assessment in the CV detection field as a reference, the performance is evaluated by the average precision, recall rate, precision rate, and *F*1 score. We determined the predicted box as positive based on a common metric where the Intersection over Union (IoU) between the predicted box and the ground truth box is greater than 0.5. The definition of the targets are

(28) $$Recall=\;\frac{X_{TP}}{X_{TP}+X_{FN}},$$

(29) $$Precision=\;\frac{X_{TP}}{X_{TP}+X_{FP}},$$

(30) $$F1\;\mathrm{score}=\;\frac{2\times Recall\times Precision}{\left(Recall+Precision\right)},$$

where Recall and Precision represent the recall and precision rate, respectively. $X_{TP}$ denotes the number of objects correctly identified as true. $X_{FP}$ denotes the number of misidentifications of false targets. $X_{FN}$ represents the number of objects that fail to be correctly detected. F1 score can be regarded as a weighted average of recall rate and precision rate to evaluate the model comprehensively. The engineering problem pays more attention to the F1 score. From the perspective of recall rate and precision rate, the experiments utilize AP to test the detection accuracy of our method.

### 3.4. Performance Comparisons

Table 1 shows the comparative results on the test set between our method and some state-of-the-art detection approaches. Faster-RCNN is a two-stage CNN-based object detector, which is a widely used non-end-to-end detection method [28]. YOLOv5n is a fast and powerful end-to-end detector and YOLOv5s denotes a larger size of YOLOv5n. YOLOv5n6 adds a detection head to YOLOv5n, which can have a larger focus scale on targets. Experiments of different color enhancement algorithms, detection structures, and YOLOs are fully taken into consideration in performance comparison.

As shown in Table 1, compared with Faster-RCNN, YOLOv5s, and YOLOv5n, the *F*1 score of YOLOv5n-EL has been enhanced by 0.148, 0.026, and 0.008, respectively. From the perspective of AP, YOLOv5n-EL achieves 0.970 AP@0.5 and 0.530 AP@0.5:0.95, which is the best of Faster-RCNN (0.832 AP@0.5 and 0.316 AP@0.5:0.95), YOLOv5s (0.957 AP@0.5 and 0.509 AP@0.5:0.95) and YOLOv5n (0.969 AP@0.5 and 0.525 AP@0.5:0.95). This illustrates the advantage of YOLOv5n-EL as a corrosion bolt detector. In this problem, the corroded bolt is the target of fixed scale, and the detection head on a larger scale may cause redundancy of features.Therefore, YOLOv5n6 failed to improve the detection performance. Meanwhile, YOLOv5n6 (0.945 AP@0.5 and 0.506 AP@0.5:0.95), which own a larger size of parameters, get a lower AP than YOLOv5n-EL. The detection time consumption of contrastive models is shown in Table 2. It is clear that the Faster-RCNN costs nearly 10 times longer than the YOLOs in experiments caused by the non-end-to-end structure. Because the features of corroded bolts in the dataset are relatively simple, the model with large parameters may be more prone to overfitting in training. In this detection task, YOLOv5n-EL not only can avoid overfitting but also achieves better performance without wasting too much time (only 7 ms more than YOLOv5n, far less than the consumption of color enhancement). Besides, the FLOPs cost of YOLOv5n-EL is still lower than YOLOv5s, while the results are significantly better. The above analysis shows the correctness of choosing YOLOv5n-EL as the detector.

With the color feature enhancement module, Table 1 also shows that MSR makes the detection performance of YOLOv5s and YOLOv5n-EL even worse instead of the enhancement. That is due to MSR causing some color distortion, which makes the data processed deviate from real data distribution. However, we notice that MSR lightly improves the detection performance of YOLOv5n6 and YOLOv5n, which illustrates that, with MSR, the overfitting caused by more parameters is somewhat relieved.

**Figure 8:**
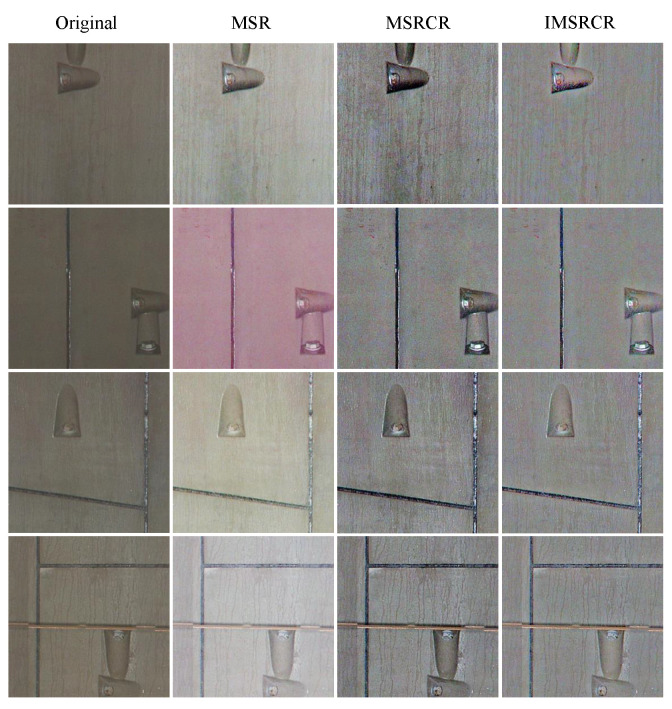
Examples of results from different color enhancement algorithm.

We also compare the results of MSRCR and IMSRCR to evaluate the performance further. It can be seen from Table 1 that, compared with MSRCR, IMRCR enhances the performance of detectors. YOLOv5n-EL achieves 0.975 mAP@0.5 and 0.537 mAP@0.5:0.95 with IMSRCR. IMSRCR effectively enhances the darker areas in the image and improves the intensity of the target edge, which offers more help to the detector. This illustrates the effectiveness of the IMSRCR method. We show the effects of different color enhancement algorithms in Figure 8. In contrast, although MSR and MSRCR can also enhance the color features of the corroded parts, color distortion may occur on other occasions, and the edge is not clear in a dim environment. The IMSRCR can not only strengthen the features significantly but also avoid obscurity in a dim environment, which leads to an improvement in comprehensive detection effectiveness.

Furthermore, Table 3 shows the time consumption of different color enhancement methods. Since MSRCR uses Gaussian blur, the enhancement speed is significantly slowed down to undertake many numerical calculations. IMSRCR, however, avoids the shortcomings, and the speed increases by about a quarter.

**Figure 9:**
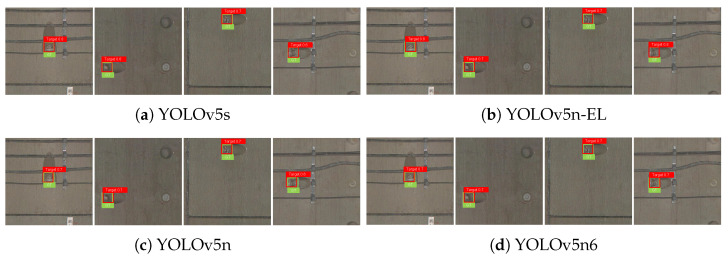
Examples of detection results without color enhancement.

**Figure 10:**
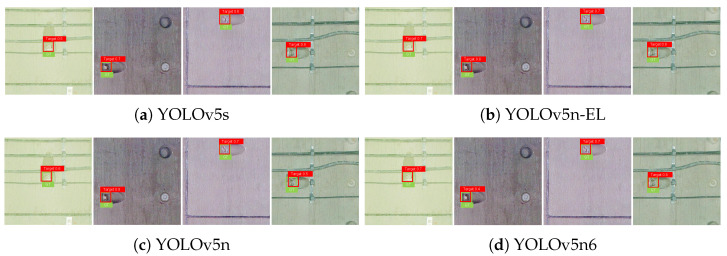
Examples of detection results with MSR color enhancement.

**Figure 11:**
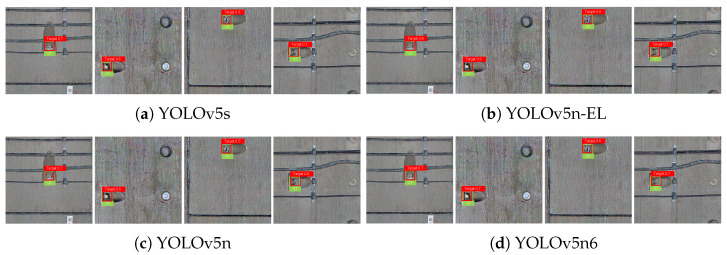
Examples of detection results with MSRCR color enhancement.

**Figure 12:**
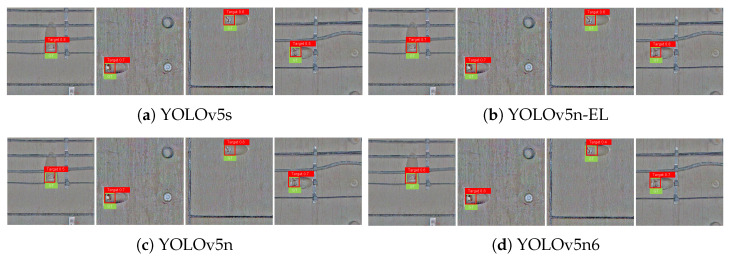
Examples of detection results with IMSRCR color enhancement.

Figure 9, Figure 10, Figure 11 and Figure 12 show the visualization of some representative detection results in the test set. Compared to other YOLO detectors and the baseline YOLOv5n, YOLOv5n-EL offers better performance in detection like Figure 9b,c. Comparison between Figure 9, Figure 10 and Figure 11 shows that different color enhancement algorithms can heighten the significance of features, changing the effect of the total model.

In summary, the experimental results indicate that YOLOv5n-EL is an efficient corroded bolt target detector. In addition, the ablation study demonstrates that the IMSRCR is helpful for the enhancement of the color features and improves the detection performance for corroded bolts both in speed and accuracy.

## 4. Discussion

The method composed in this paper is a corroded bolt detection model, which combines the IMSRCR module and YOLOv5n-EL into one algorithm. The experimental results on the test set demonstrate that our method has good detection performance for corroded bolts. The parameter size of the YOLOv5n-EL basic model (YOLOv5n) is only about 14 MB, which is suitable for project deployment and real-time detection. Our method outperforms other comparative methods in both accuracy and speed. The color feature enhancement made by IMSRCR is helpful for the detector to detect corroded bolts with inconspicuous corrosion features.

## 5. Conclusions

In this paper, a method was put forward for tunnel corroded bolt detection. For this purpose, an efficient CV module with color enhancement and ensemble learning is proposed. Considering the low definition, poor contrast, and color distortion in the tunnel, IMSRCR enhances the color and edge appearance based on auto-matched dynamic filters and $L_{0}$ regularization. Moreover, YOLOv5n-EL also directly improves the accuracy of detection. To examine the effectiveness of our model, we collect corroded bolts with a professional tunnel scanner from a practical railway tunnel. It achieves a precision of 0.921 and a recall of 0.975 within 84.237 ms (14.367 + 69.870), which confirms that the IMSRCR + YOLOv5n-EL is the most suitable structure for the task. 

## Figures and Tables

**Table 1:** The experimental results.

Method	Precision	Recall	*F*1 Score	AP@0.5	AP@0.5:0.95
Faster-RCNN	0.690	0.917	0.790	0.832	0.316
YOLOv5s	0.876	0.950	0.912	0.957	0.509
YOLOv5n6	0.883	0.927	0.904	0.945	0.506
YOLOv5n	0.889	0.974	0.930	0.970	0.525
YOLOv5n-EL (baseline)	0.895	0.974	0.938	0.970	0.530
MSR + YOLOv5s	0.886	0.924	0.905	0.959	0.510
MSR + YOLOv5n6	0.858	0.948	0.901	0.952	0.495
MSR + YOLOv5n	0.864	0.969	0.913	0.961	0.506
MSR + YOLOv5n-EL	0.880	0.970	0.922	0.965	0.515
MSRCR + YOLOv5s	0.877	0.933	0.904	0.961	0.509
MSRCR + YOLOv5n6	0.889	0.962	0.924	0.970	0.514
MSRCR + YOLOv5n	0.912	0.970	0.940	0.966	0.533
MSRCR + YOLOv5n-EL	0.917	0.970	0.943	0.972	0.534
IMSRCR + YOLOv5s	0.881	0.933	0.906	0.965	0.512
IMSRCR + YOLOv5n6	0.915	0.972	0.943	0.972	0.520
IMSRCR + YOLOv5n	0.914	0.970	0.941	0.971	0.535
IMSRCR + YOLOv5n-EL	**0.921**	**0.975**	**0.947**	**0.975**	**0.537**

**Table 2:** The Time and FLOPs Consumption of Detectors.

	Faster-RCNN	YOLOv5s	YOLOv5n	YOLOv5n-EL
FLOPs (G)	∖	15.8	4.1	12.3
Pre-process Time (ms)	∖	0.677	0.614	0.643
Inference Time (ms)	∖	9.571	5.041	12.716
NMS Time (ms)	∖	1.475	1.405	1.008
Total Time (ms)	83.990	11.723	7.060	14.367

**Table 3:** The Time Consumption of Color Enhancement.

	MSR	MSRCR	IMSRCR
Time Cost (ms)	47.576	93.308	69.870

## Data Availability

Not applicable.

## References

[1] Hu X., Cao Y., Sun Y., Tang T. (2022). Railway Automatic Switch Stationary Contacts Wear Detection Under Few-Shot Occasions. IEEE Trans. Intell. Transp. Syst..

[2] Hu X., Cao Y., Tang T., Sun Y. (2022). Data-driven technology of fault diagnosis in railway point machines: Review and challenges. Transp. Saf. Environ..

[3] Wen T., Xie G., Cao Y., Cai B. (2022). A DNN-Based Channel Model for Network Planning in Train Control Systems. IEEE Trans. Intell. Transp. Syst..

[4] Nikravesh S.M.Y., Goudarzi M. (2017). A review paper on looseness detection methods in bolted structures. Lat. Am. J. Solids Struct..

[5] Reddy M.S.B., Ponnamma D., Sadasivuni K.K., Aich S., Kailasa S., Parangusan H., Ibrahim M., Eldeib S., Shehata O., Ismail M. (2021). Sensors in advancing the capabilities of corrosion detection: A review. Sens. Actuators A Phys..

[6] Xu Y., Li D., Xie Q., Wu Q., Wang J. (2021). Automatic defect detection and segmentation of tunnel surface using modified Mask R-CNN. Measurement.

[7] Fan Z., Song Z., Xu J., Wang Z., Wu K., Liu H., He J. (2022). Object Level Depth Reconstruction for Category Level 6D Object Pose Estimation From Monocular RGB Image. arXiv.

[8] Fan Z., Liu H., He J., Jiang S., Du X. (2020). PointFPN: A Frustum-based Feature Pyramid Network for 3D Object Detection. Proceedings of the 2020 IEEE 32nd International Conference on Tools with Artificial Intelligence (ICTAI).

[9] Zhang X., Sheng Z., Shen H.L. (2022). FocusNet: Classifying better by focusing on confusing classes. Pattern Recognit..

[10] Cao S.Y., Hu J., Sheng Z., Shen H.L. Iterative Deep Homography Estimation. Proceedings of the IEEE/CVF Conference on Computer Vision and Pattern Recognition (CVPR).

[11] Chang S., Zhang R., Ji K., Huang S., Feng Z. (2022). A Hierarchical Classification Head based Convolutional Gated Deep Neural Network for Automatic Modulation Classification. IEEE Trans. Wirel. Commun..

[12] Cha Y.J., Choi W., Suh G., Mahmoudkhani S., Büyüköztürk O. (2018). Autonomous structural visual inspection using region-based deep learning for detecting multiple damage types. Comput. Aided Civ. Infrastruct. Eng..

[13] Ta Q.B., Huynh T.C., Pham Q.Q., Kim J.T. (2022). Corroded Bolt Identification Using Mask Region-Based Deep Learning Trained on Synthesized Data. Sensors.

[14] Suh G., Cha Y.J. (2018). Deep faster R-CNN-based automated detection and localization of multiple types of damage. Proceedings of the Sensors and Smart Structures Technologies for Civil, Mechanical, and Aerospace Systems.

[15] Redmon J., Divvala S., Girshick R., Farhadi A. You only look once: Unified, real-time object detection. Proceedings of the IEEE Conference on Computer Vision and Pattern Recognition.

[16] Song Y., Zhang H., Liu L., Zhong H. (2018). Rail surface defect detection method based on YOLOv3 deep learning networks. Proceedings of the 2018 Chinese Automation Congress (CAC).

[17] Guo K., He C., Yang M., Wang S. (2022). A pavement distresses identification method optimized for YOLOv5s. Sci. Rep..

[18] Xu R., Lin H., Lu K., Cao L., Liu Y. (2021). A forest fire detection system based on ensemble learning. Forests.

[19] Forkan A.R.M., Kang Y.B., Jayaraman P.P., Liao K., Kaul R., Morgan G., Ranjan R., Sinha S. (2022). CorrDetector: A framework for structural corrosion detection from drone images using ensemble deep learning. Expert Syst. Appl..

[20] Seijo-Pardo B., Porto-Díaz I., Bolón-Canedo V., Alonso-Betanzos A. (2017). Ensemble feature selection: Homogeneous and heterogeneous approaches. Knowl. Based Syst..

[21] Zhang W., Dong L., Xu W. (2022). Retinex-inspired color correction and detail preserved fusion for underwater image enhancement. Comput. Electron. Agric..

[22] Liu R., Ma L., Zhang J., Fan X., Luo Z. Retinex-inspired unrolling with cooperative prior architecture search for low-light image enhancement. Proceedings of the IEEE/CVF Conference on Computer Vision and Pattern Recognition.

[23] Fan M., Wang W., Yang W., Liu J. Integrating semantic segmentation and retinex model for low-light image enhancement. Proceedings of the ACM International Conference on Multimedia.

[24] He K., Sun J., Tang X. (2012). Guided image filtering. IEEE Trans. Pattern Anal. Mach. Intell..

[25] Ochotorena C.N., Yamashita Y. (2019). Anisotropic guided filtering. IEEE Trans. Image Process..

[26] Li Z., Song X., Chen C., Wang C. (2019). Brightness level image enhancement algorithm based on retinex algorithm. J. Data Acquisit. Process.

[27] Xu L., Lu C., Xu Y., Jia J. Image smoothing via L 0 gradient minimization. Proceedings of the 2011 SIGGRAPH Asia Conference.

[28] Ren S., He K., Girshick R., Sun J. (2015). Faster r-cnn: Towards real-time object detection with region proposal networks. Adv. Neural Inf. Process. Syst..

